# Transcriptional Dysregulation of Autophagy in Aging and Potential Interventions: Insights Into TFEB and FOXOs

**DOI:** 10.31083/FBL38730

**Published:** 2025-09-28

**Authors:** Cheng-Ju Kuo, Denisa M. Manastireanu, Jose L. Nieto-Torres, Caroline Kumsta

**Affiliations:** 1 Sanford Burnham Prebys Medical Discovery Institute, La Jolla, CA 92037, USA; 2 Department of Biomedical Sciences, School of Health Sciences, Universidad Cardenal Herrera-CEU, CEU Universities, 46115 Valencia, Spain

**Keywords:** aging/genetics, autophagy/genetics, transcription factor EB (TFEB)/microphthalmia-associated transcription factors (MITFs), forkhead box O (FOXO) transcription factors/forkhead transcription factors, drug effects/therapeutic use

## Abstract

Autophagy is a highly conserved cellular degradation and recycling process essential for maintaining cellular homeostasis. However, autophagic activity declines with age, contributing to the accumulation of damaged organelles and protein aggregates. The decline in autophagic activity is considered a primary hallmark of aging, as it contributes to cellular dysfunction and the onset of age-associated diseases, including neurodegenerative disorders and metabolic dysfunction. Sustaining autophagy with age requires transcriptional regulation, which may become impaired with age. In this review, we summarize current understanding of transcriptional regulation of autophagy during aging, with a specific focus on transcription factor EB (TFEB) and forkhead box O (FOXO) transcription factors. We integrate mechanistic insights from both mammalian systems and model organisms to highlight how their regulatory activity declines with age through changes in expression, post-translational modifications, nuclear transport, and transcriptional efficiency. We further explore pharmacological and lifestyle interventions aimed at restoring autophagic function to mitigate cellular decline. Given the pivotal role of autophagy in promoting cellular resilience and disease prevention, targeting autophagy-regulating transcription factors holds promise as a therapeutic strategy to counteract age-related functional decline and extend healthspan.

## 1. Introduction

Aging is a biological process characterized by the gradual decline in physiological functions and increased vulnerability to diseases, such as cancer, cardiovascular disorders, diabetes, and neurodegeneration [1]. Understanding aging is crucial for developing interventions to delay its progression, improve healthspan, and combat age-related diseases. The hallmarks of aging represent a set of key biological processes and mechanisms that contribute to the functional decline and increased susceptibility to diseases associated with aging [1]. These hallmarks follow three criteria: (1) the process itself declines or deteriorates with age, (2) experimentally disrupting the process accelerates the onset of aging-related features or reduces lifespan, and (3) ameliorating the impairment improves the aging-related features and extends lifespan [1].

Among these hallmarks, disabled macroautophagy (hereafter referred to as autophagy) is considered a primary hallmark of aging due to its central role in maintaining cellular homeostasis. In this review, we expand on this perspective by discussing autophagy as a hallmark of aging in greater depth, incorporating insights from model organisms that provide additional mechanistic and evolutionary context. A central contribution of this review is our indepth analysis of how transcription factor activity is modulated through changes in their expression, post-translational modifications, nuclear transport, and transcriptional efficiency. We also highlight the emerging role of transcription factors in regulating autophagy during aging, summarizing current findings and identifying key knowledge gaps on how the malfunction of these transcriptional regulators may contribute to autophagic decline over time. By integrating these perspectives, we aim to offer a novel framework for understanding autophagy decline in aging and for developing targeted strategies to mitigate age-related diseases.

## 2. Dysregulation of Autophagy as a Hallmark of Aging

The autophagy process is highly conserved from yeast to humans and is responsible for degrading and recycling diverse cytosolic cargo, including damaged organelles, protein aggregates, lipids, and pathogens [2,3]. It is controlled by upstream regulators like the mechanistic target of ra- pamycin complex 1 (mTORC1), which inhibits autophagy under nutrient-rich conditions, and AMP-activated protein kinase (AMPK), which activates it during energy stress. Upon activation, the Autophagy-Related Gene 1/Unc-51-Like Kinase 1 (ATG1/ULK1) complex (ATG13, FAK family-interacting protein of 200 kDa (FIP200), ATG101) initiates phagophore formation by phosphorylating and activating the BECN1/Vacuolar Protein Sorting 34 class III phosphatidylinositol 3-kinase (BECLIN1/VPS34 class III PI3K complex), which drives vesicle nucleation. The elongation of the autophagosome membrane relies on two major ubiquitin-like conjugation systems: the ATG12-ATG5-ATG16/ATG16L1 complex and ATG7-dependent lipidation of Autophagy-Related Gene 8/Microtubule-Associated Proteins 1A/1B Light Chain 3 (ATG8/LC3). ATG7, an E1-like enzyme, plays a crucial role in conjugating ATG12 to ATG5, which then associates with ATG16L1 to facilitate membrane expansion. Simultaneously, ATG7 and ATG3 mediate the lipidation of ATG8 (LC3 in mammals), converting LC3-I to LC3-II, which integrates into the autophagosomal membrane [4]. During this process, cytosolic components are sequestered into double-membrane vesicles called autophagosomes. The mature autophagosome then fuses with lysosomes through Ras-related protein Rab-7 (RAB7), Soluble NSF Attachment Protein Receptors (SNARE proteins), including Syntaxin 17, Synaptosomal-associated protein 29 (SNAP29), Vesicle-associated membrane protein 8 (VAMP8), as well as Lysosome-associated membrane proteins 1 and 2 (LAMP1/LAMP2), forming an autolysosome where lysosomal enzymes, such as cathepsins, degrade its contents, alleviating intracellular proteotoxic stress and providing essential building blocks to support cellular adaptation during proteotoxic insults, nutritional stress or infections [5]. Thus, autophagy functions as a crucial quality-control mechanism that prevents the accumulation of cellular damage, a key feature of cellular aging.

Several lines of evidence support the classification of disabled autophagy as a primary hallmark of aging, as it meets the three defining criteria. First, autophagy activity diminishes with age across various organisms. For example, transcript levels of autophagy genes *Atg2*, *Atg8a*, and *Atg18* decline in the brain of the fruit fly *Drosophila melanogaster* [6]. Similarly, genome-wide microarray analysis in human brains shows a significant downregulation of autophagy mRNA in aging samples, including several key components such as *ATG7*, *ATG5*, and *ATG16L2* [7]. Furthermore, the levels of two crucial autophagy proteins, LC3-II and ATG7, are found decreased in the muscle [8] and hypothalamus [9] of aging mice and also in human muscles [8]. Rubicon, a negative regulator of autophagy increases in expression with age across species, including *C. elegans*, *Drosophila*, and mice [10].

Second, experimental inhibition of autophagy accelerates aging and reduces lifespan. For instance, loss or reduction of autophagy shortens lifespan in worms, flies, and mice [6,8,11]. In mice, muscle-specific knockout of *Atg7* reduces lifespan [8], paralleling findings in the nematode *Caenorhabditis elegans*, where muscle-specific inhibition of the autophagy genes *lgg-1/Atg8* or *atg-18* reduces the lifespan of long-lived insulin/IGF-1 signaling mutants (*daf-2*/insulin/insulin-like growth factor 1 receptor) [12].

Notably, suppression of the negative autophagy-regulator Rubicon, has been shown to restore autophagy, extend lifespan in *C. elegans* and *Drosophila*, and ameliorate age-related diseases such as Parkinson’s disease and age-related macular degeneration in mice [10,13]. This finding leads into the third criterion of a hallmark of aging: interventions that enhance autophagic activity, by genetic or pharmacological means, improve aging-related features and extend lifespan. Genetic activation of autophagy, either through *Atg5* overexpression improves motor function and healthspan [14], while a BECLIN1 (F121A) mutation that constitutively activates autophagy increases basal autophagic flux, prolongs lifespan, and improves healthspan in mice [15], demonstrating that sustained autophagy induction can delay age-related pathologies. In adult *Drosophila*, lifespan can also be extended through neuronal and muscular overexpression of Atg8 [6,16], or via neuron-specific overexpression of Atg1 [17]. Moreover, in *C. elegans* and *Drosophila*, overexpression of the selective autophagy receptor p62 orthologs, *Sequestosome-1* in *C. elegans* (SQST-1) and *Refractory to Sigma P* (Ref(2)P) in *Drosophila*, has been linked to enhanced lifespan [18,19], highlighting how the targeted degradation of specific cellular cargo, i.e., protein aggregates and mitochondria, contributes to longevity. Pharmacological approaches, such as supplementation with spermidine, an autophagy inducer, extend longevity in worms, flies [20], and mice [21]. Similarly, rapamycin, the mTORC1 inhibitor, enhances autophagy and has been shown to extend lifespan in model organisms from yeast to mice [22]. Urolithin A, a compound derived from dietary polyphenols, induces mitophagy, and improves muscle function in aged mice [23]. In humans, urolithin A improves aerobic endurance and muscle strength in middleaged adults [24] and reduces neuroinflammation in Parkinson’s disease patients [25]. Other pharmacological interventions, such as metformin and resveratrol, have also been shown to promote autophagy and extend lifespan in model organisms, highlighting the potential of autophagy enhancement as a strategy to slow aging and prevent age-related diseases [26].

Autophagy is not only essential for quality control, metabolic adaptation, and cellular defense but also intersects with other hallmarks of aging. For example, autophagy mitigates mitochondrial dysfunction, proteostasis imbalance, and chronic inflammation, thereby preventing the onset or exacerbation of these hallmarks. As autophagy declines with age, its protective functions are compromised, creating disruptions of additional hallmarks. Autophagy’s ability to influence and regulate multiple aging mechanisms underscores its pivotal role in maintaining cellular health and preventing age-related decline. However, the precise mechanisms driving the age-related decline in autophagic activity remain incompletely understood despite their critical contribution to aging and associated diseases.

Emerging evidence underscores the critical role of transcriptional regulation in modulating autophagy during aging, since transcription factors integrate upstream cellular and environmental signals to govern autophagic activity [27–29]. Dysregulation of these pathways has been implicated in the decline of autophagic capacity, with transcription factors such as transcription factor EB (TFEB) and forkhead box O proteins (FOXOs), which are key regulators of autophagy-related genes, exhibiting altered activity with age [16,18]. Understanding how transcriptional regulation contributes to autophagy during aging is crucial, as these represent promising targets for therapeutic interventions aimed at restoring autophagic function and mitigating age-related diseases [28,30,31]. This review explores the current knowledge on transcriptional regulation of autophagy in aging, as well as the treatments that may boost autophagy via transcriptional regulation, aiming to highlight their potential as therapeutic targets to promote healthy aging.

## 3. Mechanisms of Transcriptional Regulation of Autophagy

The induction of autophagy is governed by both post-translational and transcriptional mechanisms [32]. The initial induction of autophagy occurs rapidly, within minutes of exposure to stress [33,34] and can persist for several weeks [35]. The immediate activation relies on post-translational modifications of existing proteins [36–38], while sustained autophagy requires the activation of autophagy-related genes to produce new proteins, a process mediated by transcription factors, to ensure the continued functionality of autophagy over time [39]. Beyond transcription factor activity, autophagy is also regulated by epigenetic mechanisms and non-coding RNAs (ncRNAs), which modulate gene accessibility and expression. Epigenetic regulation via DNA methylation, chromatin remodeling, and histone post-translational modifications, such as methylation and acetylation, directly impacts the chromatin structure, without altering DNA sequence, thereby modulating the accessibility of transcription factors to DNA and gene expression. Notably, transcription factors and epigenetic modifications interact through a complex, interdependent network that finely controls autophagy and lysosomal gene activity. The broader impacts of epigenetic modifications on autophagy regulation, which go beyond the primary scope of this review, have been discussed elsewhere [40–42]. Likewise, ncRNAs, including microRNAs (miRNAs) and long ncRNAs (lncRNAs), regulate autophagy through multiple mechanisms. miRNAs typically interfere with autophagy gene mRNA levels, while lncRNAs can influence chromatin remodeling or directly modify the activity of autophagy proteins. In fact, growing literature reviews the profound impact of ncRNAs on autophagy regulation in detail [43,44]. Although chromatin-level and post-transcriptional regulation contribute to autophagy-gene transcription changes, transcription factors remain the primary executors of stress- and stimulus-responsive autophagy gene programs. These transcription factors integrate upstream inputs, such as nutrient availability, hypoxia, endoplasmic reticulum (ER) and heat stress, as well as DNA damage, to directly regulate autophagy-related gene expression. In the following section, we provide a brief overview of select stress-responsive transcription factors that have been implicated in autophagy regulation, before focusing on two critical regulators, TFEB and FOXO proteins, whose activity is tightly linked to aging and therapeutic potential.

Several transcription factors mediate autophagy in response to cellular stress [32]. For example, the ER stress-responsive factor X-box-binding protein 1 (XBP1) directly binds to the *BECLIN1* promoter to activate autophagy [45]. Activating transcription factor 4 (ATF4) promotes autophagy under ER stress or hypoxia by binding to the *ULK1* promoter [46], as demonstrated through chromatin immunoprecipitation (ChIP) analysis. Hypoxia-inducible factor 1*α* (HIF-1*α*) enhances autophagy under hypoxic conditions by activating multiple autophagy-related genes [47–49]. Nuclear factor-kappa B (NF-*κ*B) is required for heat shock-induced autophagy and supports cell survival following heat stress in human cells [50]. Additionally, ChIP-seq and RNA-seq analyses in mouse embryo fibroblasts have also revealed that p53 directly regulates several autophagy-related genes, including *Ulk1, Atg2, Atg4, Atg7*, and *Atg10* as well as select lysosomal genes, particularly in the context of DNA damage [51]. Interestingly, while nuclear p53 promotes autophagy, in human cells, a cytoplasmic form of p53 lacking a nuclear localization sequence can inhibit autophagy [52].

While these transcription factors clearly play key roles in stress-induced autophagy, relatively little is known about how their activity is affected by aging or whether they contribute to age-related changes in autophagy. This remains an important area for future investigation. In contrast, this review focuses on two evolutionarily conserved transcription factors, TFEB and FOXO family proteins, whose sustained dysregulation has been directly implicated in autophagy decline during aging. Both act as master regulators of autophagy and lysosomal gene expression and respond to nutrient and stress signals in diverse organisms. Their central roles in maintaining cellular homeostasis, promoting stress resilience, and regulating lifespan position them as promising targets for restoring autophagy function and mitigating age-related pathologies.

### 3.1 Transcription Factor EB (TFEB)

**Fig. 1.**
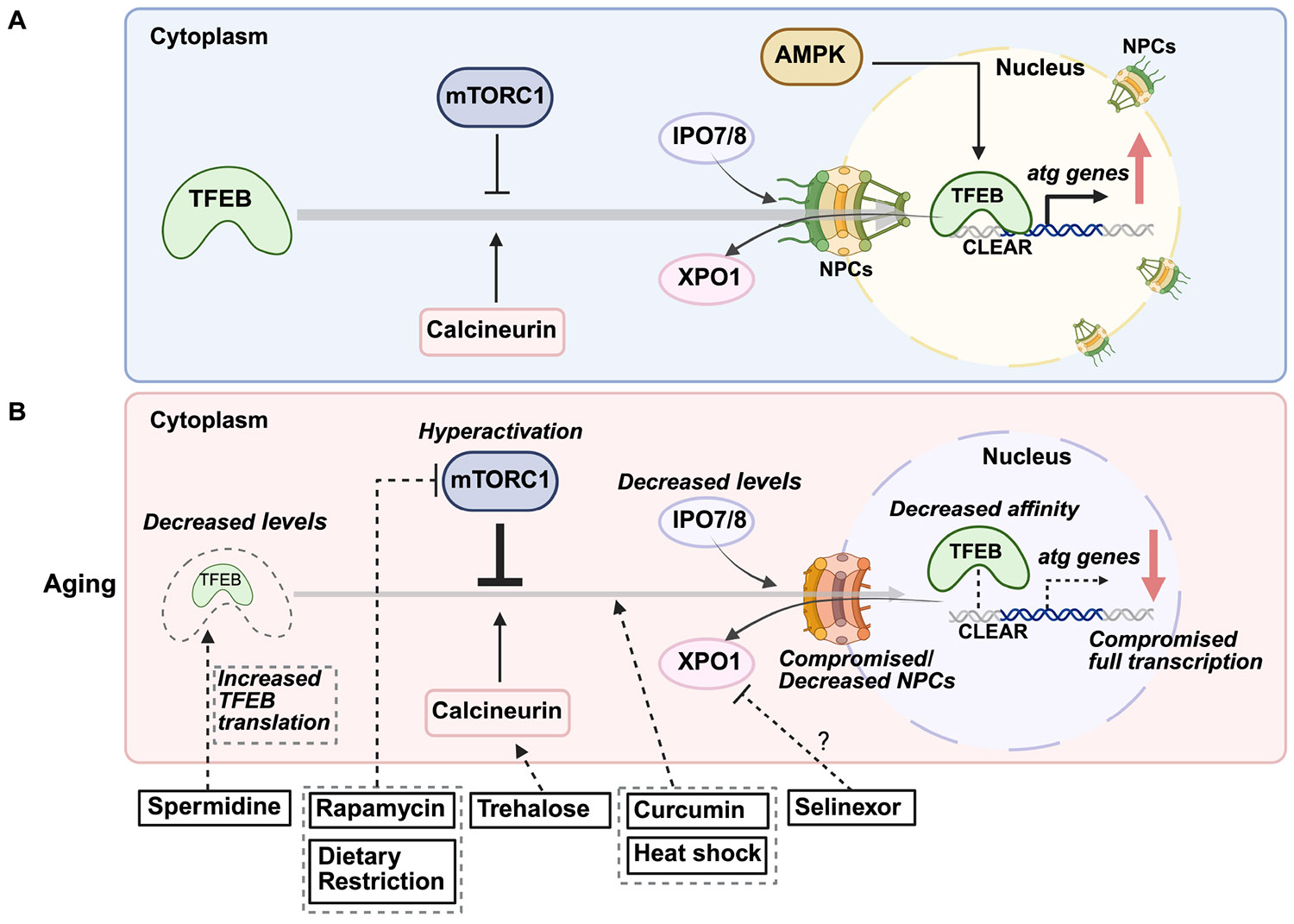
Age-related changes in transcription factor EB (TFEB) regulation and therapeutic interventions. (A) Mechanisms regulating TFEB nuclear localization. Under basal conditions, the mechanistic target of rapamycin complex 1 (mTORC1) phosphorylates TFEB, preventing its entry into the nucleus and confining it to the cytoplasm. In response to cellular stress, calcineurin (CaN) dephosphorylates TFEB, facilitating its nuclear import and promoting autophagy. AMP-activated protein kinase (AMPK) also enhances TFEB activity by phosphorylation. The nuclear import of TFEB relies on transport via importins (IPO7/8) through nuclear pore complexes (NPCs). Once cellular stress is resolved, TFEB is exported back to the cytoplasm via binding with the exportin 1 (XPO1)/chromosome region maintenance 1 (CRM1). (B) Age-associated decline in TFEB and potential therapeutic strategies. Dysregulation of upstream regulators may alter TFEB post-translational modifications, impairing its nuclear translocation. Additionally, TFEB protein levels decline with age, reducing the transcription of autophagy-related genes. Age-related impairments in nucleocytoplasmic transport, possibly due to reduced NPC/importin expression and/or NPCs impairment, may further disrupt TFEB localization. Potential interventions: Rapamycin inhibits mTORC1, relieving TFEB repression and promoting autophagy. Trehalose activates CaN, likely via lysosomal calcium release, facilitating TFEB nuclear translocation. Spermidine enhances TFEB protein levels by promoting its translation. Curcumin prevents TFEB sequestration in the cytoplasm, ensuring its nuclear retention and autophagy activation. Selinexor, an XPO1/CRM1 inhibitor, may restrict TFEB within the nucleus, sustaining autophagy. Dietary restriction enhances TFEB nuclear translocation by suppressing mTORC1 activity. Heat shock has been shown to promote TFEB nuclear localization, further supporting autophagy. CLEAR, coordinated lysosomal expression and regulation.

TFEB is recognized as a primary regulator of autophagy- and lysosomal-gene expression and is a member of the microphthalmia-associated transcription factor (MITF) family [53]. TFEB binds to the coordinated lysosomal expression and regulation (CLEAR) motifs (GTCACGTGAC) in target gene promoters, driving the expression of genes involved in autophagy and lysosomal biogenesis. TFEB activity is tightly regulated by nutrient and stress signaling pathways. Under normal, nutrientrich conditions, TFEB is phosphorylated by mTORC1 at serine residues 122, 142, and 211 (post-translational modifications data refer to human proteins unless otherwise indicated). This phosphorylation promotes its binding to 14-3-3 proteins, masking its nuclear localization signal (NLS) and resulting in cytoplasmic retention and suppression of its transcriptional activity [28]. The folliculin (FLCN) complex, in association with FLCN-interacting proteins 1 and 2 (FNIP1/2), and the RAG GTPases Ras-related GTP-binding protein A/D (RAG A/D) and Ras-related GTP-binding protein C/D (RAG C/D), further facilitate mTORC1 localization to the lysosome, maintaining TFEB phosphorylation and inhibition [54]. In response to stress, such as nutrient deprivation, mTORC1 activity decreases, and TFEB undergoes dephosphorylation, liberating it from 14-3-3 proteins [55]. This process is mediated by calcineurin (CaN), a calcium-dependent phosphatase activated by lysosomal calcium release through channels such as transient receptor potential mucolipin 1 (TRPML1) and two-pore channels (TPCs), which respond to changes in lysosomal lipid composition and ion gradients [56]. Additionally, AMPK directly enhances TFEB activity by phosphorylation serine residues 466, 467, and 469, thereby increasing its transcriptional capacity [57]. Dephosphorylation of TFEB at S142 and S211 promotes its nuclear translocation, where it activates genes essential for autophagy and lysosomal function [57] (Fig. 1A).

Nuclear transport receptors, importin 7 (IPO7) and importin 8 (IPO8), recognize TFEB’s unmasked NLS and assist its transport into the nucleus [58]. Once there, TFEB binds to CLEAR motifs in promoter regions of autophagy and lysosomal genes, driving transcriptional upregulation. Meanwhile, in the cytoplasm, the chaperone-dependent E3 ubiquitin ligase Stress-Induced Phosphoprotein 1 (STIP1) homology and U-Box containing protein 1 (STUB1) binds to phosphorylated, inactive TFEB (S142 and S211), targeting it for proteasomal degradation [59]. This regulation controls TFEB stability and facilitates nuclear translocation. When cellular stress subsides and nutrient levels are restored, mTORC1 has been proposed to re-phosphorylate TFEB at serine residues 138 and 142 within the nucleus [60]. This posttranslational modification exposes a nuclear export signal (NES) on the TFEB N-terminus, allowing interaction with the nuclear export receptor exportin 1 (XPO1) (also known as chromosome region maintenance 1, CRM1), facilitating TFEB’s export from the nucleus to the cytoplasm [60]. This finely tuned regulatory network allows TFEB to respond dynamically to cellular stress. However, age-related dysregulation, including mTORC1 hyper-activation, impaired calcium signaling, and reduced TFEB levels, can disrupt this balance, contributing to diminished autophagic capacity in aged cells [61,62] (Fig. 1A).

### 3.2 Forkhead Box O Proteins (FOXOs)

**Fig. 2.**
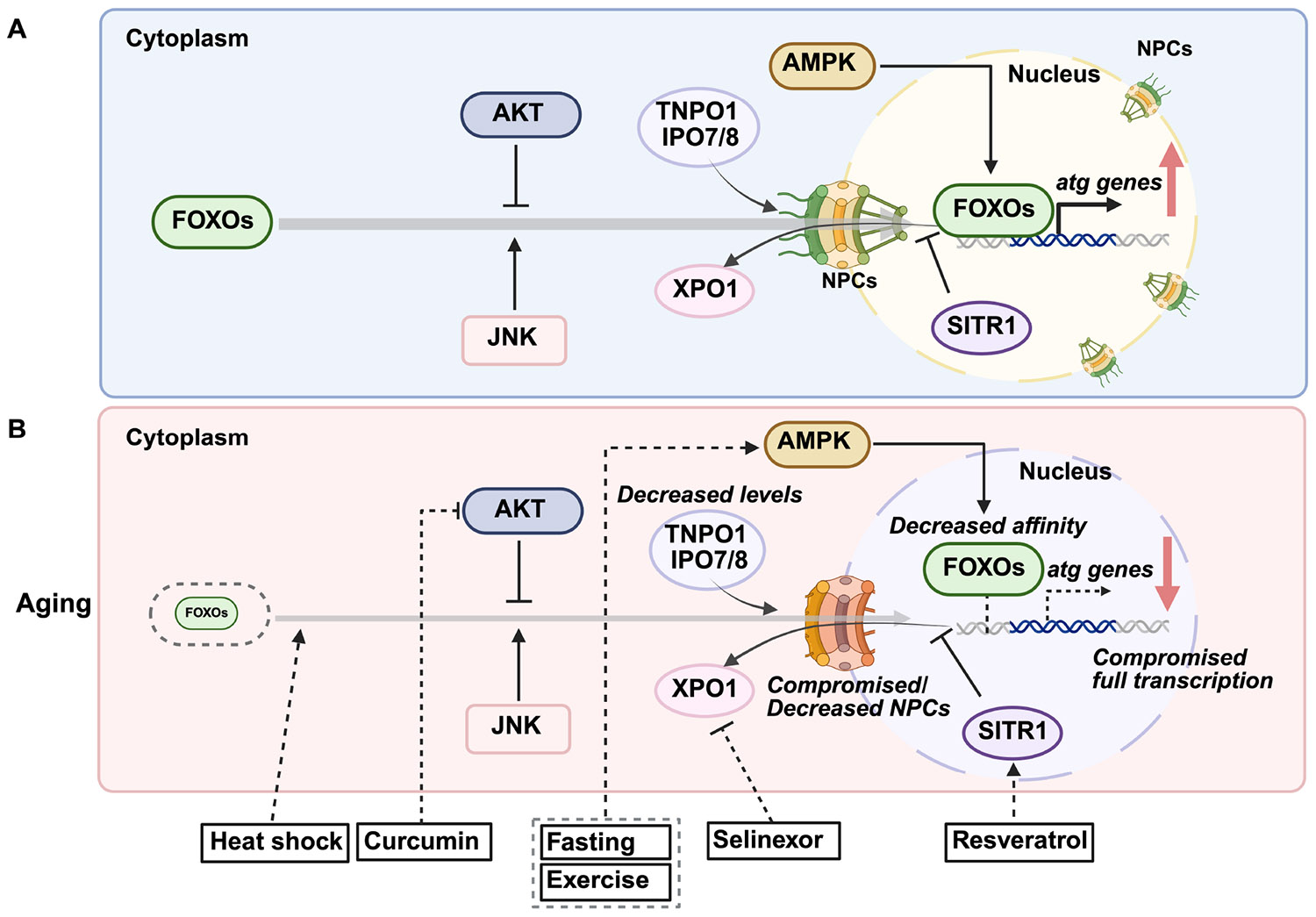
Age-related changes in forkhead box O proteins (FOXOs) regulation and therapeutic interventions. (A) Mechanisms governing FOXOs nuclear translocation. Under basal conditions, FOXO transcription factors are phosphorylated by the protein kinase B (AKT) pathway, preventing their nuclear entry and retaining them in the cytoplasm. During cellular stress, Jun N-terminal kinase (JNK) phosphorylates FOXOs at distinct sites, promoting their nuclear translocation and activation. Additionally, AMP-activated protein kinase (AMPK) phosphorylates FOXOs, enhancing their transcriptional activity. The nuclear import of FOXOs depends on transportin TNPO1 and importins IPO7/8 to facilitate their passage through nuclear pore complexes (NPCs). Once stress conditions subside, FOXOs are exported back to the cytoplasm through binding with the XPO1. (B) Age-related decline in FOXOs and potential interventions. FOXO protein levels may decrease over time, reducing the transcription of autophagy-related genes. Additionally, age-related defects in nucleo-cytoplasmic transport, possibly due to diminished NPC/importin expression and/or NPCs impairment, can further impair FOXO nuclear localization. The overall efficiency of autophagy gene transcription may decline with age, further compromising cellular proteostasis. Several interventions have been identified to enhance FOXO activity and counteract age-related autophagic decline: Fasting and exercise activate AMPK, leading to FOXO phosphorylation and autophagy induction. Curcumin inhibits PI3K-AKT-mTOR signaling to promote autophagy. Resveratrol activates sirtuin 1 (SIRT1), which deacetylates FOXOs, promoting their nuclear retention. Selinexor, an XPO1/CRM1 inhibitor, restricts FOXOs within the nucleus, sustaining autophagy. Heat shock has been shown to enhance FOXO nuclear localization, further supporting autophagic activity.

In addition to TFEB, another major family of transcription factors regulating autophagy is FOXO. Similar to TFEB, the ability of FOXOs to induce autophagy-related genes is regulated by posttranslational modifications that change their subcellular localization. Under normal conditions, FOXOs are phosphorylated by the phosphatidylinositol 3-kinase/protein kinase B (PI3K/AKT) pathway activated in response to growth factor or insulin signaling. These phosphorylation events at two serines and one threonine (S256/253, S319/315, and T24/32 in FOXO1 and FOXO3, respectively), create binding sites for 14-3-3 proteins, which sequester FOXOs in the cytoplasm, preventing their nuclear localization by masking their nuclear import signals [63]. Serum/Glucocorticoid Regulated Kinase (SGK) can also phosphorylate FOXOs at these sites, exerting a similar inhibitory effect on FOXO function [64]. In addition to inhibitory phosphorylations, FOXOs are activated by stress-responsive post-translational modifications. For instance, increased reactive oxygen species (ROS) activate Jun N-terminal kinase (JNK), which phosphorylates FOXO3 at S294, disrupting their binding to 14-3-3 proteins. This releases FOXO3 for nuclear translocation and transcriptional activation of autophagy-related genes [65]. Similarly, AMPK phosphorylates FOXO3 at S413 and S588 under nutrient deprivation, enhancing its transcriptional activity without affecting its localization or DNA-binding ability [63,66]. Acetylation further modulates FOXO activity, generally acting as an inhibitory modification by reducing DNA-binding affinity and altering sensitivity to AKT phosphorylation. In FOXO1, CREB-binding protein (CBP), a histone acetyltransferase, induces acetylation at K242 and K245 within the DNA-binding domain, decreasing transcriptional activity [67]. Additionally, acetylation at K222, K245, K248, K262, K265, K274, and K294 has been reported to influence its interaction with AKT signaling [68,69]. Conversely, oxidative stress-induced deacetylation enhances FOXO transcriptional activation, promoting the expression of autophagy-related genes [68]. When AKT activity is inhibited, AKT-mediated phosphorylations are presumably removed by protein phosphatase 2A (PP2A), liberating FOXO proteins from 14-3-3 binding and allowing their nuclear accumulation [70]. With age, disruptions in stress-signaling pathways, alterations in FOXO post-translational modifications, and reduced FOXO activity can contribute to declined autophagic efficiency [63,71] (Fig. 2A).

## 4. FOXO–TFEB Crosstalk in Autophagy and Longevity Regulation

Notably, *C. elegans* studies reveal that the FOXO ortholog *Abnormal Dauer Formation-16* (DAF-16) and the TFEB ortholog *Helix-Loop-Helix family member 30* (HLH-30) share functional overlap in regulating autophagy and aging-related processes. Knockdown of *daf-16* shortens the lifespan of both wild-type and long-lived *daf-2*/insulin/IGF1 signaling mutants, mirroring the effects observed with *hlh-30* silencing [72]. Moreover, HLH-30/TFEB and DAF-16/FOXO co-regulate genes involved in oxidative stress responses, proteolysis, and the unfolded protein response [72]. In mammalian systems, there is also evidence of direct regulatory interaction. FOXO1 binds to the *TFEB* promoter in murine adipocytes, promoting *TFEB* transcription [73]. Pharmacological inhibition of FOXO1 reduces *TFEB* transcript and protein levels, leading to diminished autophagy [73]. This complex regulatory network ensures a coordinated response to nutrient and stress signals, optimizing autophagy and promoting organismal longevity.

## 5. Age-Related Dysregulation of Autophagy Transcription Factors and Disease Implications

How does aging affect transcription factors, leading to the dysregulation of autophagy? This dysregulation may be linked to age-related changes in transcription factor expression. Interestingly, both age-related decreases and increases in transcription factor abundance have been described, which seem to be species-, tissue- and context-specific. In mammals, aging does not uniformly decrease autophagy-related transcription factors; instead, their regulation varies by factor and tissue. For example, in aged mice, TFEB protein levels decline in both cytosolic and nuclear fractions of hippocampal and cortical tissues [61]. Additionally, in murine and human B cells, levels of spermidine, a polyamine that promotes TFEB expression and autophagic activity, decrease with age [74], suggesting an age-related reduction in TFEB levels. The regulation of FOXO transcription factors (FOXO1, FOXO3, and FOXO4) during aging is tissue-dependent. In aged male rats, FOXO3 mRNA levels decline in skeletal muscles, whereas the levels of another isoform, FOXO1, are not affected [75]. Similarly, in aged mice, FOXO3 protein levels decrease in the cortex but remain unchanged in the hippocampus [76]. However, another study found that FOXO transcription factors (FOXO1, FOXO3, and FOXO4) progressively increase in transcript and protein expression in the aging human and mouse brain [77], suggesting a potential compensatory role in response to age-related cellular stress. This upregulation may be neuroprotective, as neuron-specific deletion of FOXOs leads to age-related axonal degeneration, impaired autophagy, elevated proteotoxic stress, and motor dysfunction in mice [77]. Moreover, FOXO deficiency impairs autophagic flux in developing neurons of the adult mouse hippocampus, leading to altered dendritic morphology, increased spine density, and abnormal spine positioning [78]. These findings suggest that FOXOs contribute to neuronal health during aging, and their dysregulation may increase vulnerabilities to neurodegenerative conditions. Overall, these findings highlight species- and tissue-dependent differences in transcription factor regulation with aging, which may contribute to the complex, context-specific decline of autophagy.

In *C. elegans*, single-cell RNA sequencing (scRNA-seq) reveals an age-associated increase in *TFEB/hlh-30* and *FOXO3/daf-16* mRNA in most cell types [79]. Despite this increase in mRNA levels, autophagic activity appears blocked with age in most *C. elegans* tissues [12]. This suggests that aging may trigger a compensatory mechanism aimed at upregulating stress-responsive transcription factors to counteract age-related cellular dysfunction. However, rather than restoring autophagy, this response may result in a dysregulated transcriptional landscape, with most *atg* genes being upregulated while some are suppressed [79].

While altered transcription factor levels with age can affect autophagy, their proper function also depends on nuclear translocation, which may be impaired during aging (Fig. 1B). For example, TFEB must translocate to the nucleus to activate autophagy-related genes, a process tightly regulated by mTORC1 signaling. With age, abnormal cytoplasmic retention of TFEB, potentially due to hyperactivation of mTORC1 signaling, may impair its function. mTORC1 phosphorylates TFEB, preventing its nuclear translocation. Increased mTORC1 activity has been observed in aged muscle tissues in mice [80] and rats [81], where its overactivation is implicated in conditions such as sarcopenia [81,82], while in neurons, it is commonly linked to neurodegenerative disorders, including Alzheimer’s disease [83,84]. Conversely, calcineurin (CaN), a phosphatase activated by cellular stress, dephosphorylates TFEB to promote nuclear translocation [85]. Age-related declines in CaN function may further impair the nuclear localization of TFEB, although CaN hyperactivity has been reported in aged and Alzheimer’s brains [86], suggesting distinct regulatory mechanisms (Fig. 1B). This raises the possibility that mTORC1-driven cytoplasmic retention of TFEB represents the predominant mechanism, or that CaN hyperactivity alone is insufficient to counteract mTORC1 hyperactivation. Moreover, in patients with Alzheimer’s disease, decreased expression and impaired nuclear localization of TFEB have been observed in blood cells [87] and brains [88]. In the midbrain of Parkinson’s disease patients, TFEB is also abnormally retained in the cytoplasm, likely due to *α*-synuclein aggregation [89], with similar TFEB sequestration and autophagy impairment observed in rat models expressing human *α*-synuclein [89].

The nuclear import of large proteins, such as transcription factors, requires strict regulatory mechanisms. Transcription factors enter the nucleus via nuclear pore complexes (NPCs), which act as selective gates embedded in the nuclear envelope, controlling access to genetic material [90]. Large molecules rely on importins, transport receptors that serve as “keys” for NPC entry [90]. With age, declines in nucleocytoplasmic transport efficiency may hinder transcription factor nuclear entry and impair autophagy regulation. In human diploid fibroblasts, importin levels are notably reduced in the nuclear fractions of senescent cells, indicating age-related downregulation [91] (Fig. 1B). Additionally, in postmitotic cells like neurons and somatic cells of *C. elegans*, NPCs deteriorate with age, leading to nuclear pore leakiness [92]. While similar mechanisms have been observed in induced neurons (iNs) from aged donors [93], their full extent in the aged human brains remains to be fully explored. These changes could limit transcription factor nuclear localization, ultimately impairing autophagy. However, there is currently no direct evidence linking age-related impairment of nucleocytoplasmic transport to disrupted autophagy-related transcription factor nuclear translocation, highlighting the need for further research. Such studies could reveal therapeutic targets to enhance the nuclear translocation of transcription factors to combat age-related autophagy decline.

Finally, aging may impair the transcriptional efficiency of transcription factors, thereby decreasing the expression of autophagy-related genes. ChIP-seq analysis shows that FOXO binding affinity to target gene promoters significantly decreases in aged female *Drosophila* [94]. This could be due to altered post-translational modifications of FOXO, changes in co-transcriptional regulation between FOXO and its partners or changes in epigenetic landscape (Fig. 2B). Additional experiments are required to understand how aging affects the capacity of TFEB and FOXOs to engage the transcription of autophagy-related genes. Most likely, the molecular bases of such changes will be connected to changes in the epigenetic marks, which usually cooperate with transcription factors to regulate gene expression and are known to be influenced by aging. In addition, once started, age-related decline of mRNA transcription proficiency could affect TFEB and FOXO targets. A recently proposed concept, gene-length-dependent transcription decline [95,96], suggests that aging disproportionately affects the transcription of longer genes, possibly due to RNA polymerase stalling caused by accumulated DNA damage [97]. This phenomenon has been observed in postmitotic cells across species, from nematodes to humans. Although autophagy-related genes are not specifically characterized by long gene lengths, their transcription may still be affected indirectly through age-related impairments in the broader transcriptional machinery. Further investigation into how aging impacts TFEB and FOXO’s transcriptional capacity could provide additional insights into autophagy regulation.

Collectively, these findings underscore the multifaceted regulation of transcription factors and their vulnerability to age-related changes at the transcriptional, translational, post-translational, and functional levels. Future studies should explore whether restoring nucleocytoplasmic transport or enhancing transcription factor binding affinity could mitigate age-related autophagy decline, particularly in the context of age-related diseases.

## 6. Potential of Autophagy-Regulating Transcription Factors as Therapeutic Targets

Multiple pharmacological and lifestyle interventions have been identified that modulate autophagy by targeting key transcription factors, including TFEB and FOXOs (Fig. 1B and Fig. 2B). These strategies aim to counteract the age-related decline in autophagic activity and offer therapeutic potential for age-associated diseases [31,98]. Among these, several compounds have been shown to enhance autophagic activity by activating TFEB and FOXOs.

### 6.1 Pharmacological Strategies

Spermidine, a polyamine and known autophagy inducer, extends lifespan across various organisms, including yeast, worms, flies, and mice [20], and reduces Alzheimer’s disease-associated amyloid-beta levels in mice [99]. In humans, dietary supplementation of spermidine correlates with reduced cardiovascular disease incidence, improved cognitive function [21], and enhanced lifespan in epidemiological studies [100]. These effects are partly mediated by the hypusination of eukaryotic translation initiation factor 5A (eIF5A) [74], which increases TFEB protein levels and restores impaired autophagy. Additionally, spermidine inhibits histone acetyltransferases (HAT) in yeast, reducing histone H3 acetylation and inducing autophagic gene transcription [20,101]. Rapamycin, an mTORC1 inhibitor that indirectly activates TFEB by inhibiting its cytoplasmic retention via mTORC1 [102], activates autophagy and extends lifespan in yeast [103], worms [104], flies [105], and mice [106]. While these studies confirmed autophagy induction, they did not assess TFEB nuclear localization or activity. Rapamycin also attenuates age-related sarcopenia by partially inhibiting overactivated mTORC1 [81,82]. Additionally, rapamycin demonstrates therapeutic potential in models of Alzheimer’s, Parkinson’s, and Huntington’s diseases [107], and improves vascular and cardiac functions [108–110]. Trehalose, a naturally occurring disaccharide, activates CaN, likely via lysosomal calcium release, promoting TFEB nuclear translocation and enhancing autophagy [111], which has demonstrated neuroprotective effects [112]. Although trehalose does not directly regulate FOXOs, FOXO3/DAF-16 drives trehalose production in organisms such as *C. elegans*, where it supports adaptation to starvation [113], and may contribute to the induction of autophagy under starvation conditions. Curcumin, a natural polyphenolic compound, and its analogs induce autophagy by directly interacting with TFEB at the N-terminus, enhancing its nuclear translocation, likely by reducing 14-3-3 binding [114,115]. These compounds show potential in ameliorating neurodegenerative and cardiovascular diseases [116]. Curcumin also promotes autophagy via FOXO1, as its ability to induce autophagy under oxidative stress is lost when FOXO1 expression is knocked down [117]. Other autophagy-inducing interventions act via FOXO-dependent pathways. Resveratrol, a natural polyphenol, activates FOXO transcription factors through multiple mechanisms. It inhibits the PI3K/AKT signaling pathway, reducing FOXO phosphorylation and promoting its nuclear translocation [118]. Additionally, resveratrol activates sirtuin 1 (SIRT1), a NAD^+^-dependent deacetylase, which deacetylates FOXO proteins, enhancing their DNA-binding ability and transcriptional activity [119]. Resveratrol has been shown to enhance autophagic flux via the SIRT1/FOXO1/Rab7 axis, leading to improved cardiac function and reduced oxidative injury in diabetic mice [120]. Additionally, resveratrol increases the expression of autophagy-related genes such as *LC3B* and B-cell lymphoma 2 (BCL2) interacting protein 3 (BNIP3) via FOXO activation, contributing to cardioprotection in models of Duchenne muscular dystrophy [121].

While enhancing autophagic activity through TFEB and FOXO activation holds therapeutic potential for aging-related diseases, in cancer, transcription factor modulation has a dual role: FOXO activation can promote tumor suppression, whereas TFEB inhibition may help restrict autophagy-dependent tumor survival. Selinexor, a synthetic XPO1/CRM1 inhibitor, has been clinically approved for the treatment of multiple myeloma [122] and has been shown to promote nuclear retention of FOXO1 [123], thereby restoring its transcriptional activity to induce cell cycle arrest and apoptosis of cancer cells. Inhibition of XPO1/CRM1 also leads to the nuclear retention of TFEB/HLH-30 [60,124], and while the effect of selinexor on TFEB has not yet been explored sustained TFEB activation may support tumor growth by enhancing autophagy, making its inhibition a more desirable strategy in certain cancers. One such example is eltrombopag, a small molecule thrombopoietin receptor agonist and the first identified direct inhibitor of TFEB [125]. Eltrombopag binds to TFEB’s basic helix-loop-helix-leucine zipper domain, disrupting its interaction with DNA. This compound effectively inhibits starvation-induced autophagy and, as a Food and Drug Administration (FDA)-approved drug, has potential as a therapeutic agent for autophagy-dependent cancers [125]. A summary of the chemical compounds targeting TFEB and FOXOs is presented in Table 1 (Ref. [74,102,111,114,115,117–119,122,123,125]).

### 6.2 Lifestyle Interventions

In addition to pharmacological approaches, lifestyle interventions such as dietary restriction and exercise have shown potential in inducing autophagy and delaying aging [4] (Fig. 1B and Fig. 2B). Dietary restriction activates autophagy through nutrient-sensing pathways, including AMPK and mTORC1, which converge on transcription factors like TFEB and FOXOs to regulate autophagy-related genes [126]. Similarly, regular exercise enhances autophagic flux in skeletal muscle, contributing to improved metabolic health and resilience against age-related decline [127]. Another promising autophagy-inducing intervention is heat stress. Regular sauna use (2–3 sessions per week at 80–100 °C) is associated with reduced cardiovascular disease, dementia, and all-cause mortality [128,129], although direct evidence of sauna-induced autophagy in humans is limited. In mammals, heat shock increases autophagy markers such as BECLIN1 and LC3-II in mouse myotubes [130], along with elevated p62 mRNA levels in human peripheral blood mononuclear cells (PBMCs) [131]. In model organisms like *C. elegans*, heat stress activates autophagy, extends lifespan, and improves proteostasis through nuclear translocation of TFEB/HLH-30 [132] and FOXO3/DAF-16 [133], as well as through activation of heat-shock factor heat shock transcription factor 1 (HSF-1) [132]. When and how these stress-activated transcription factors co-regulate autophagy under stress and during aging is not fully understood. Considering that FOXO/DAF-16 and TFEB/HLH-30 have been reported to co-regulate several target genes [72], these results suggest that FOXO3/DAF-16 may also play a role in regulating autophagy under heat stress. Further investigation is warranted to understand how heat shock mitigates age-related dysregulation of transcription factors to promote autophagy.

In summary, aging affects multiple aspects of TFEB and FOXOs function, potentially contributing to the decline in autophagic capacity observed in various tissues. Understanding how aging alters TFEB and FOXOs protein levels, subcellular localization, nucleocytoplasmic transport, and transcriptional efficiency could offer valuable insights into the mechanisms of age-related autophagy decline and reveal therapeutic targets for age-associated diseases.

## 7. Conclusions

Autophagy plays a critical role in maintaining cellular homeostasis, particularly in postmitotic cells, such as neurons and cardiomyocytes, which cannot undergo proliferation to remove or dilute harmful components, such as protein aggregates, through cell division. These cells thus heavily rely on proteasomes (a protein complex that degrades ubiquitinated proteins) and lysosomes to manage proteotoxic stress. Additionally, different cell types may exhibit varying rates of aging, leading to distinct requirements for stress response mechanisms. Tissue-specific declines in autophagic activity have been observed in *C. elegans*, and autophagy is required in distinct tissues in two longevity paradigms (reduced *daf-2*/insulin/IGF1 signaling and germline-less mutants), highlighting its tissue-specific roles [12]. In mammals, skeletal muscle, liver, and brain exhibit differential patterns of autophagy decline with age [134], emphasizing tissue-dependent autophagy regulation. Furthermore, specific age-related diseases correlate with autophagy decline. Impaired autophagy is strongly linked to neurodegenerative disorders such as Alzheimer’s and Parkinson’s disease, as well as age-related metabolic diseases like sarcopenia, type 2 diabetes, and cardiovascular diseases [135]. Understanding how aging affects autophagy regulation and the differential autophagic demands of various cell types is essential for developing targeted therapies to mitigate age-related autophagic decline and associated diseases.

Given the central role of transcription factors in sustaining a proficient autophagy pathway, it is tempting to argue that their age-dependent dysregulation may represent a key driver of autophagy decline. Conversely, this also makes them attractive targets for therapeutic interventions aimed at modulating autophagy during aging. A range of interventions, including heat shock, dietary restriction, intermittent fasting, and exercise are known to induce autophagy and attenuate aging, in part through transcriptional regulation. In addition, pharmacological approaches such as rapamycin (mTORC1 inhibition), spermidine (enhanced TFEB translation), trehalose (TFEB nuclear translocation), and curcumin (FOXO activation) offer promising strategies to restore autophagic activity. However, the efficacy and safety of these interventions may be highly context-dependent. Notably, TFEB and FOXO transcription factors, while promoting autophagy and cellular clearance, can act as either tumor suppressors or tumor promoters depending on cellular context [136]. In some scenarios, their activation helps prevent tumor initiation by maintaining proteostasis and reducing oxidative damage [136,137]. In contrast, in established tumors, enhanced autophagy can support cancer cell survival under metabolic stress, contributing to progression and therapy resistance [138]. Therefore, activating FOXOs or TFEB may carry context-dependent risks, particularly in cancer-prone tissues. In sum, autophagy is like a double-edged sword: it provides protection when properly regulated, but can become harmful if it is overactivated or dysregulated. Therapeutic strategies should aim to fine-tune autophagy and its tissue/organ-dependent regulation, enhancing its beneficial effects (such as protein clearance and stress adaptation) while minimizing adverse outcomes like tumor support or excessive degradation of essential cellular components.

Future research should address several open questions. It will be important to investigate the molecular mechanisms behind the failure of the autophagy-related transcription factors, including the novel implications of their nucleocytoplasmic transport processes. Accordingly, whether restoration of their nuclear import mechanisms can rescue autophagic decline should be tested. Furthermore, understanding how FOXOs and TFEB are regulated and interact in a tissue-specific manner may reveal new insights into transcriptional networks governing autophagy in aging. Additionally, determining how to activate these transcription factors selectively in aging tissues without promoting survival in malignant cells remains a critical challenge. Overall, an integrated approach to studying autophagy regulation in aging is needed that incorporates transcriptional, post-transcriptional, and post-translational mechanisms. Notably, there remains a substantial gap in human aging studies that directly investigate transcriptional regulation of autophagy. A recent review in this Special Issue discusses human-relevant models for studying autophagy in aging, including primary cells and iPSC-derived systems, and outlines current limitations and opportunities for translation [139]. Finally, future studies should also explore whether sex-specific differences influence autophagy decline or the efficacy of interventions, particularly in relation to transcription factor regulation, as current evidence remains limited. Given autophagy’s fundamental role in cellular homeostasis and longevity, therapies aimed at restoring TFEB and FOXO function hold strong promise to mitigate age-related functional decline and extend healthspan.

## Figures and Tables

**Table 1.** Summary of the chemical interventions targeting TFEB and FOXOs.

Transcription factors	Compound	Interventions	References
TFEB	Spermidine	Increases TFEB protein levels	[74]
	Rapamycin	Inhibits mTORC1, relieving TFEB cytoplasmic retention	[102]
	Trehalose	Activates CaN to promote nuclear translocation	[111]
	Curcumin	Enhances TFEB nuclear retention by reducing 14-3-3 binding	[114,115]
	Eltrombopag	Inhibits TFEB DNA binding, suppressing transcription	[125]
FOXOs	Curcumin	Inhibits PI3K-AKT-mTOR signaling to promote activation	[117]
	Resveratrol	Promotes nuclear retention via SIRT1-mediated deacetylation and AKT inhibition	[118,119]
	Selinexor	Inhibits XPO/CRM1 to enhance FOXO nuclear retention	[122,123]

## Abbreviations

AKT: protein kinase B
AMPK: AMP-activated protein kinase
ATF4: activating transcription factor 4
ATG: autophagy-related gene
BCL2: B-cell lymphoma 2
CBP: CREB-binding protein
CaN: calcineurin
CLEAR: coordinated lysosomal expression and regulation
CRM1: chromosome region maintenance 1
DAF-16: abnormal dauer formation-16
eIF5A: eukaryotic translation initiation factor 5A
FIP200: FAK family interacting-protein of 200 kDa
FOXO: forkhead box O
HLH-30: helix-loop-helix family menber 30
HSF-1: heat shock transcription factor 1
IGF1: insulin-like growth factor 1
IPO7: importin 7
IPO8: importin 8
JNK: Jun N-terminal kinase
LAMP: Lysosomal-associated membrane protein
LC3: microtubule-associated proteins 1A/1B light chain 3
MITF: microphthalmia-associated transcription factor
mTORC1: mechanistic target of rapamycin complex 1
NF-*κ*B: nuclear factor-kappa B
NPC: nuclear pore complex
PBMCs: peripheral blood mononuclear cells
PI3K: phosphatidylinositol 3-kinase
PP2A: protein phosphatase 2A
RAB7: Ras-related protein Rab-7
RAG: Ras-related GTP-binding protein
Ref(2)P: Refractory to Sigma P
SIRT1: sirtuin (silent mating type information regulation 2 homolog) 1
SNARE: soluble N-ethylmaleimide-sensitive factor attachment protein recep-tor
SNAP29: synaptosome-associated protein 29
SQST-1: sequestosome-1
STIP1: stress-induced phosphoprotein 1
STUB1: STIP1 homology and U-box containing protein 1
TPCs: two-pore channels
TRPML1: transient receptor potential mucolipin 1
ULK: Unc-51-like kinase
VAMP8: vesicle-associated membrane protein 8
XBP1: X-box-binding protein 1
XPO1: exportin 1.
